# Clinical efficacy comparison of CO2 laser treatment and LEEP surgery for cervical intraepithelial neoplasia with high-risk HPV infection

**DOI:** 10.4314/ahs.v25i2.8

**Published:** 2025-06

**Authors:** Shanshan Huang, Hongling Guo, Tingting Yu, Guojian Gu, Xiaofa Wu, Xiang Li, Yan Cheng

**Affiliations:** 1 Department of Gynecology, Taicang Affiliated Hospital of Soochow University, Taicang First People's Hospital, Taicang, China; 2 Department of Pathology, Taicang Affiliated Hospital of Soochow University, Taicang First People's Hospital, Taicang, China; 3 Department of General Practice, Taicang Affiliated Hospital of Soochow University, Taicang First People's Hospital, Taicang, China

**Keywords:** CO2 laser, loop electrosurgical excision procedure, cervical intraepithelial neoplasia, high-risk human papillomavirus, clinical efficacy, safety

## Abstract

**Background:**

To investigate the clinical efficacy of CO2 laser and loop electrosurgical excision procedure (LEEP) in the treatment of cervical intraepithelial neoplasia (CIN) with high-risk human papillomavirus (HPV) infection.

**Methods:**

A total of 150 CIN patients with high-risk HPV infection admitted to our hospital from December 2020 to June 2022 were divided into CO2 laser treatment group (CO2 group) with 63 cases and LEEP treatment group (LEEP group) with 87 cases based on treatment principles and patient preferences. The postoperative recovery time, CIN treatment effect, high-risk HPV clearance rate, treatment safety, and recurrence rate were compared between the two groups.

**Results:**

The vaginal discharge time, vaginal bleeding time, and wound healing time in the CO2 group were significantly lower than those in the LEEP group (P < 0.05). The incidence of adverse events in the CO2 group was 14.29%, which was significantly lower than that in the LEEP group (28.74%, P < 0.05).

**Conclusion:**

CO2 laser and LEEP surgery for the treatment of CIN combined with high-risk HPV infection both have good clinical efficacy, and CO2 laser can effectively shorten the postoperative recovery time with good safety.

## Introduction

Cervical intraepithelial neoplasia (CIN) is a process in which the squamous epithelium of the cervix progresses from a normal structure to a tumor, closely associated with persistent infection of high-risk human papillomavirus (HPV). It is currently a well-established important pathogenic factor of cervical cancer^1^,^2^. CIN can be divided into high-grade squamous intraepithelial lesion (HSIL) or low-grade squamous intraepithelial lesion (LSIL). Studies have shown that patients with persistent LSIL are at higher risk of developing cervical cancer and require early non-invasive intervention^3^,^4^. Currently, the clinical practice often uses loop electrosurgical excision procedure (LEEP) to treat LSIL/CIN patients, which can quickly remove the diseased tissue with good efficacy using high-frequency electric waves. However, there is a risk of postoperative cervical adhesion or cervical stenosis caused by cervical matrix injury in some patients^5^,^6^. CO2 laser surgery can localize carbonize, necrosis, and shed the diseased tissue by a strong pulse to form new tissue to restore normal cervical tissue, avoiding cervical excision with high safety and stability^7^. Previous studies have reported on the efficacy analysis of LEEP in treating CIN^8^, but there are few reports on comparative analysis of the efficacy of LEEP and CO2 laser treatment in patients with CIN and concurrent high-risk HPV infection. This study mainly explores the clinical efficacy of CO2 laser and LEEP surgery in the treatment of CIN and concurrent high-risk HPV infection, aiming to provide reference for clinical treatment decisions for patients with CIN and concurrent high-risk HPV infection.

## Patients and Methods

### Patients

We selected a total of 150 patients with cervical intraepithelial neoplasia (CIN) and high-risk human papillomavirus (HPV) infection, who were admitted to the First People's Hospital of Taicang from December 2020 to June 2022 (https://www.chictr.org.cn/showproj.html?proj=176602: Clinical trial no. ChiCTR2200063068).

Inclusion criteria: 1. Patients who met the diagnostic criteria for CIN: This refers to the presence of abnormal cellular changes in the squamous epithelium of the cervix, as confirmed by histopathological examination; 2. Positive high-risk HPV-DNA detection: Patients who tested positive for high-risk HPV DNA using specific laboratory tests; 3. Histopathological examination indicating low-grade squamous intraepithelial lesions (LSIL): The histopathological examination of cervical tissue samples showed the presence of LSIL, which is a form of CIN characterized by mild cellular abnormalities; 4. Ability to tolerate surgery: Patients who were deemed medically fit to undergo surgical intervention based on their overall health status and absence of contraindications.

Exclusion criteria: 1. Presence of malignant cells or suspected cancerous lesions: Patients who showed evidence of malignant cells or suspected cancerous lesions based on cytological or histological examination were excluded from the study; 2. Inability to rule out invasive cancer: Patients for whom invasive cancer could not be ruled out through vaginal colposcopy examination were excluded; 3. Severe pelvic inflammation, cervical inflammation, or other severe gynecological inflammations: Patients with severe inflammatory conditions in the pelvic or cervical region, or other severe gynecological inflammations, were excluded; 4. Pregnancy or lactation: Pregnant or lactating patients were excluded from the study due to potential risks associated with the surgical interventions and the impact on maternal and fetal health; 5. Presence of other malignant tumors: Patients with a concurrent diagnosis of other malignant tumors were excluded to maintain homogeneity within the study population. Patients were divided into two groups according to treatment principles and patient preference: 63 patients underwent CO2 laser treatment (CO2 group) and 87 patients underwent loop electrosurgical excision procedure (LEEP group). This study was approved by the Ethics Committee of the First People's Hospital of Taicang (Y-21-082).

### Treatment Methods

For patients with cervical intraepithelial neoplasia (CIN) and high-risk human papillomavirus (HPV) infection in the LEEP group, LEEP knife treatment was performed. The procedure was performed 3-7 days after the end of menstruation, and preoperative tests included cervical liquid-based cytology examination, HPV testing, and pathological biopsy under vaginal colposcopy. The patient was placed in a lithotomy position, and the external genitalia and vagina were routinely disinfected. The range of the surgery was determined by performing an iodine test on the cervical surface. After local anesthesia was administered to the external genitalia and cervix, a dilator was used to fully expose the cervical opening, and an appropriate electric knife was selected to remove the lesion. For patients with CIN1, the depth of the surgical resection was 1.5 cm, and the range of resection extended 1 mm beyond the iodine test area. For patients with CIN2, the depth of the surgical resection was 1.5-2.0 cm, and the range of resection extended 3 mm beyond the iodine test area. After the excised tissue was marked and sent for pathological examination, the pathological investigation included an examination of the edge of the lesion tissue.

For patients with CIN and high-risk HPV infection in the CO2 group, CO2 laser treatment was performed. The preoperative preparation was the same as for the LEEP group. After cleaning the external genitalia and inserting a vaginal speculum, the CO2 laser treatment device was used to vaporize the lesion in continuous output mode. The laser wavelength was set to 10.6 µm, the power was set to 25 W, and the spot diameter was 1-3 mm. The lesion was treated in a layer-by-layer manner in a clockwise direction from the outer edge 5 mm away from the lesion, and the treatment distance from the lesion tissue was 3-5 cm, with a treatment depth of 6-7 mm. After the treatment was completed, the wound was washed with physiological saline.

### Outcome Measures

#### Postoperative recovery time

We recorded the time to vaginal discharge, vaginal bleeding, and wound healing in two groups of patients with CIN and high-risk HPV infection. Vaginal discharge and bleeding time were defined as the time from the appearance of yellow secretion and bleeding in the vagina to complete cessation, and wound healing time was defined as the time from the end of surgery to the time when the vaginal mucosa appeared smooth during follow-up under vaginal microscopy.

#### Clinical efficacy^10^

We conducted gynecological examinations, cytological examinations, HPV virus detection, and vaginal microscopy in the first to sixth month after surgery to compare the effectiveness of CIN treatment and the conversion rate of high-risk HPV to negative between the two groups of patients with CIN and high-risk HPV infection. CIN treatment effectiveness was defined as the absence of lesions on vaginal microscopy and histological diagnosis during follow-up after surgery. The conversion rate of high-risk HPV to negative was defined as consecutive negative results for high-risk HPV-DNA on HPV testing during follow-up, indicating the conversion of high-risk HPV to negative.

#### Safety Evaluation

We recorded the incidence of adverse events such as local tissue necrosis, shedding, lower back pain, cervical adhesions, increased vaginal discharge, and cervical canal scar stenosis during the treatment period in the two groups of patients with CIN and high-risk HPV infection.

#### Follow-up and Recurrence Rate

Both groups with combined CIN and high-risk HPV infection underwent an 8-month follow-up after surgery (Note: Some patients remained HPV-positive at the 6-month follow-up). Follow-up assessments included gynecological examination, cytology examination, HPV virus detection, and vaginal colposcopy to observe patient recurrence. Recurrence^11^ was defined as no lesions found on vaginal colposcopy within 8 months after treatment, but lesions were found again on colposcopy and biopsy 8 months after treatment completion.

### Statistical analysis

Statistical Product and Service Solutions (SPSS) 22.0 software (IBM, Armonk, NY, USA) was used for data analysis. Continuous variables (such as postoperative recovery time for both groups with combined CIN and high-risk HPV infection) were expressed as mean ± standard deviation (s), and differences were compared using t-tests. Categorical variables (such as the effective rate of CIN treatment for both groups with combined CIN and high-risk HPV infection) were expressed as percentages, and differences were compared using χ2 or Fisher's exact tests. A p-value of less than 0.05 was considered statistically significant.

## Results

### Comparison of baseline data between the two groups of CIN patients with high-risk HPV infection

There was no significant difference in age, CIN grade and gravidity between the two groups in CIN patients with high-risk HPV infection (P > 0.05). See Table 1.

**Table 1:** Comparison of baseline data between the two groups of CIN patients with high-risk HPV infection [n (%), x̅±s]

Group	Number of cases	Age (years)	CIN Grade (I/II)	Gravidity (times)
CO_2_ group	63	41.93 ± 3.78	25/38	1.45 ± 0.23
LEEP Group	87	42.05 ± 3.64	42/45	1.44 ± 0.27
*t/χ* ^2^		0.196	1.092 1.092	0.237
*P*		0.845	0.296	0.812

Comparison of postoperative recovery time between the two groups of CIN patients with high-risk HPV infection

Vaginal discharge time, vaginal bleeding time, and wound healing time were significantly lower in the CO 2 group than in the LEEP group (P < 0.05). See Table 2.

**Table 2:** Comparison of postoperative recovery time between the two groups of CIN patients with high-risk HPV infection (x̅±s)

Group	Number of cases	Vaginal discharge time (d)	Vaginal bleeding time (d)	Wound healing time (d)
CO_2_ group	63	12.52 ± 1.98	12.29 ± 1.34	11.62 ± 1.28
LEEP Group	87	15.86 ± 2.23	15.78 ± 2.65	14.19 ± 2.04
*t*		9.483	9.596	8.817
*P*		0.000	0.000	0.000

Comparison of CIN treatment effect between the two groups of CIN patients with high-risk HPV infection The effective rate of CIN treatment in CO 2 group was 25.40%, 49.21%, and 68.25% at 1, 3, and 6 months after operation, respectively, which was not significantly different from 32.18%, 54.02%, and 72.16% in LEEP group (P > 0.05). See Table 3.

**Table 3:** Comparison of response rate of CIN treatment between the two groups [n (%)]

Group	Number of cases	1 month post-op	3 months after surgery	6 months post-op
CO_2_ group	63	16 (25.40)	31 (49.21)	43 (68.25)
LEEP Group	87	28 (32.18)	47 (54.02)	70 (72.16)
*X* ^2^		0.812	0.340	0.282
*P*		0.368	0.560	0.596

Comparison of high-risk HPV negative rate between the two groups in CIN patients with high-risk HPV infection

There was no significant difference in high-risk HPV negative conversion rate at 1 month, 3 months and 6 months after operation in CIN patients with high-risk HPV infection in CO 2 group (P > 0.05). See Table 4.

**Table 4:** Comparison of high-risk HPV negative rate between the two groups [n (%), x̅±s]

Group	Number of cases	High-risk HPV negative rate [n (%)]		

		1 month post-op	3 months after surgery	6 months post-op
CO_2_ group	63	10 (15.87)	40 (63.49)	47 (64.38)
LEEP Group	87	14 (16.09)	47 (54.02)	65 (74.71)
*t*		0.001	1.345	2.017
*P*		0.971	0.246	0.156

Safety evaluation of treatment in patients with CIN complicated with high-risk HPV infection in two groups None of the CIN patients with high-risk HPV infection in the CO 2 group developed cervical canal scar stenosis, and the incidence of adverse events such as local tissue necrosis and shedding, backache, cervical adhesion, and increased leucorrhea in CIN patients with high-risk HPV infection in the CO 2 group was 14.29%, which was significantly lower than 28.74% in the LEEP group (P < 0.05). See Table 5.

**Table 5:** Safety evaluation of treatment in two groups of CIN patients with high-risk HPV infection [n (%)]

Group	Number of cases	Local tissue necrosis and shedding	Backache	Cervical adhesion	Increased leucorrhea	Total AE Rate
CO_2_ group	63	5 (7.94)	2 (3.17)	1 (1.59)	1 (1.59)	9 (14.29)
LEEP Group	87	11 (12.64)	5 (5.75)	3 (3.45)	6 (6.91)	25 (28.74)
*X* ^2^						4.363
*P*						0.037

### Follow-up and Recurrence Rate

There were 5 recurrences in CIN patients with high-risk HPV infection in the CO 2 group and 2 recurrences in the LEEP group, and the recurrence rates of CIN patients with high-risk HPV infection in the two groups were 7.94% and 2.30%, respectively, The difference was not statistically significant (Fisher P = 0 .131).

## Discussion

Cervical intraepithelial neoplasia (CIN) is closely associated with cervical cancer and is caused by repeated stimulation or chronic inflammation of the cervix, with atypical squamous cells present. When high-risk HPV infection is present, cervical biopsy and tissue excision are required to prevent disease progression^12^,^13^. Currently, clinical treatment for CIN with high-risk HPV infection includes surgical and physical therapies, with loop electrosurgical excision procedure (LEEP) commonly used in surgical treatment of HSIL/CIN and LSIL/CIN, and CO2 laser treatment most commonly used in physical therapy for LSIL/CIN^14^. This study mainly compares and analyzes the clinical efficacy of CO2 laser and LEEP in treating LSIL/CIN with high-risk HPV infection.

The results of this study showed that the vaginal drainage time, vaginal bleeding time, and wound healing time of the CO2 group were significantly lower than those of the LEEP group, indicating that CO2 laser treatment for CIN with high-risk HPV infection can effectively shorten the postoperative recovery time for these patients. CIN patients may experience slight redness and exudation from the wound 1-3 days after surgery, and crusts may fall off around 7 days post-surgery, leading to increased vaginal discharge. Additionally, ruptured local capillaries during treatment can cause persistent vaginal bleeding. The use of a hot knife in LEEP surgery can cause thermal damage to normal cervical tissue, leading to the formation of a large amount of yellow secretion and bleeding due to crusts falling off after surgery^15^. However, CO2 laser can coagulate, burn, and cut the target tissue using a 10.6 µm gas laser wavelength, causing less thermal damage to normal tissue and effectively protecting the normal cervical tissue around the lesion, thereby achieving the goal of shortening the postoperative vaginal drainage time, vaginal bleeding time, and wound healing time^16^.

In this study, the effective rates of CIN treatment in the CO2 group at 1, 3, and 6 months after surgery were 25.40%, 49.21%, and 68.25%, respectively. These rates were not significantly different from those in the LEEP group, which were 32.18%, 54.02%, and 72.16%, respectively. Furthermore, the rates of high-risk HPV clearance at 1, 3, and 6 months after surgery were also not significantly different between the two groups. These results suggest that CO2 laser treatment and LEEP surgery have similar efficacy in treating CIN and promoting high-risk HPV clearance in patients with HPV infection.

Previous studies have shown that factors affecting high-risk HPV clearance include HPV subtypes, viral load, immune status, and treatment methods. LEEP surgery, which involves using an electric knife to remove lesions under vaginal colposcopy, allows for sufficient exposure of cervical lesions, facilitating the complete removal of cervical tissue with lesions and effectively reducing the degree of HPV infection^17^. However, some studies have indicated that CO2 laser treatment, while effective in exposing cervical lesions, may be more easily affected by vaginal mucosal folds, making it difficult to clear all lesions in one treatment and leading to the possibility of postoperative HPV persistent infection in some patients^18^. Nonetheless, both treatment methods used in this study demonstrated good lesion and high-risk HPV clearance effects for patients with CIN and HPV infection, resulting in good clinical efficacy.

The reason for this may be attributed to the CO2 laser used in this study, which has advantages such as a small divergence angle, high energy density, and deep tissue penetration. Its depth of action is only 0.1 mm, which allows for precise control of cutting depth and accurate location of lesion tissue for effective clearance of lesions, achieving therapeutic and HPV clearance effects that are equivalent to those of LEEP surgery.

Our study results showed that the incidence of adverse events, such as local tissue necrosis and shedding, lower back pain, fever, and increased vaginal discharge, was significantly lower in the CO2 group compared to the LEEP group in patients with CIN combined with high-risk HPV infection. Moreover, there was no significant difference in the recurrence rate between the two groups during the 6-month follow-up period. These findings suggest that CO2 laser treatment is safe for patients with CIN combined with high-risk HPV infection and does not affect the recurrence rate. The specific analysis indicates that the use of continuous output mode to vaporize the lesion during CO2 laser treatment effectively clears local lesions in patients with CIN combined with high-risk HPV infection and helps to reduce the risk of postoperative recurrence. Some studies suggest that CO2 laser treatment is more effective for LSIL/CIN patients, while LEEP surgery is still necessary for HSIL/CIN patients^19^. Other studies have proposed that while LEEP can accurately provide relevant specimens on the basis of effectively clearing diseased tissue, CO2 laser treatment is a destructive therapy that cannot provide tissue specimens for pathological examination. Therefore, vaginal colposcopy is still necessary before surgery, and there may be risks of cervical adhesions and cervical canal scar stenosis after the procedure. Close monitoring of adverse events is required after CO2 laser treatment to assess its effectiveness^20^,^21^. In addition, the reasons why there were no statistically significant differences in the effective rate of CIN treatment, high-risk HPV conversion rate, and recurrence rate between the two groups may be due to the small sample size and short observation time, which may lead to sampling bias. This is also one of the limitations of our study, and further large-scale, multicenter studies with longer observation times and larger sample sizes are needed to verify our findings.

This study has several limitations that should be acknowledged. Firstly, the sample size was relatively small, limiting the generalizability of the findings. Additionally, being a single-center study, the results may not be applicable to broader populations, and multi-center studies are needed for validation. The relatively short follow-up period might not capture long-term outcomes and recurrence rates accurately. Furthermore, the allocation of patients to treatment groups based on treatment principles and patient preferences introduces selection bias. The lack of randomization and potential confounding factors could affect the comparability of the two treatment modalities. These limitations highlight the need for larger, randomized, multi-center studies with longer follow-up periods to provide more robust evidence.

## Conclusion

In summary, both CO2 laser and LEEP surgery have demonstrated good clinical efficacy and high clearance rates for high-risk HPV infections in the treatment of CIN. Specifically, CO2 laser treatment can effectively shorten patient recovery time without increasing the risk of recurrence and with good safety.
